# The myth of rectovaginal septum endometriosis

**DOI:** 10.1530/RAF-25-0164

**Published:** 2026-01-05

**Authors:** M Leonardi, M Marien, S J Dedden, G Condous, S M Freger

**Affiliations:** ^1^ Department of Obstetrics and Gynecology, McMaster University, Hamilton, Canada; ^2^ Department of Gynecology, Amphia Hospital, Breda, Netherlands; ^3^ The University of Sydney, Nepean Clinical School, Kingswood, Sydney, New South Wales, Australia

**Keywords:** endometriosis, terminology, rectovaginal septum

## Abstract

Endometriosis is a chronic condition where tissue resembling the uterine lining grows elsewhere in the pelvis, often causing pain, infertility, and inflammation. For decades, textbooks and research papers have described the rectovaginal septum (RVS), the thin layer between the vagina and rectum, as a common site of deep endometriosis. Yet this belief has never been conclusively proven. In our study of 161 patients, combining advanced ultrasound, meticulous keyhole surgery, and tissue analysis, we found no evidence of endometriosis within the RVS. Instead, all lesions were located in surrounding structures, including the uterosacral ligaments, the rectouterine pouch, or the bowel. These findings challenge a long-standing anatomical misconception and call for the retirement of outdated terminology. Clarifying where endometriosis truly occurs is critical for improving diagnosis, surgery, and communication in women’s health. Our study overturns decades of dogma by showing that ‘rectovaginal septum endometriosis’ may be a myth.

## Introduction

**Figure 1:**
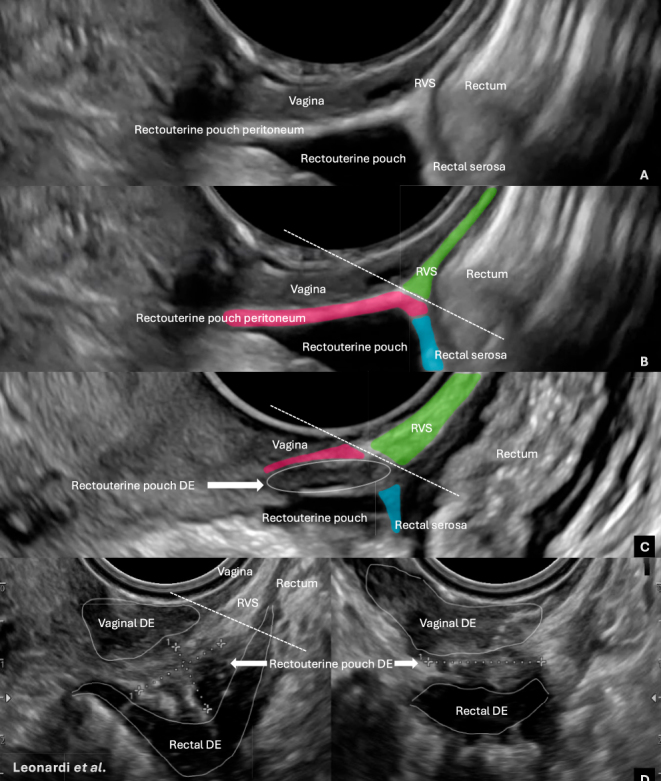
Sonographic depiction of the posterior compartment anatomical structures, normal (A and B) and with DE (C and D). (A) Normal anatomy without colour overlays depicting the vagina (hypoechoic band closest to the ultrasound probe), rectouterine pouch peritoneum overlying the vagina (hyperechoic thin tissue band), rectovaginal septum (hyperechoic band, contiguous with rectouterine pouch peritoneum, between the vagina and retroperitoneal rectum), rectum (mixed echogenic features with various layers), and overlying intraperitoneal hyperechoic rectal serosa. (B) Matching normal anatomy with colour overlays. (C) Non-obliterated rectouterine pouch (i.e. no adhesions), with DE within the rectouterine pouch peritoneum, above and at the peritoneal reflection, but without infiltration into the RVS. (D) Severe posterior compartment DE with three distinct nodules within the vagina, rectouterine pouch peritoneum, again above and at the peritoneal reflection, and intraperitoneal rectum. The adhesions, i.e. obliteration between the rectum and the vagina via the rectouterine pouch peritoneum, give the impression that the central DE lesion is within the retroperitoneum. Understanding the division between the RVS and the peritoneal reflection (even when obliteration exists) permits the understanding that the RVS is essentially preserved in severe posterior compartment endometriosis cases.

Endometriosis is a chronic inflammatory disease often causing pain and infertility (International Working Group of AAGL, ESGE, ESHRE and WES *et al.* 2021). The IDEA group has aimed to overcome longstanding diagnostic and terminology inconsistencies (Guerriero *et al.* 2016), particularly regarding deep endometriosis (DE) in the rectovaginal septum (RVS), historically reported in up to 52% of cases (Guerriero *et al.* 2008). The 2016 Cochrane review further illustrates the lack of consensus, presenting multiple definitions of RVS involvement and heterogeneous diagnostic accuracy (Nisenblat *et al.* 2016). The RVS is a retroperitoneal space (Fig. 1) long described as a common DE site; however, this interpretation is frequently confounded by severe adhesions in the rectouterine pouch (RUP) that obscure the peritoneal reflection. Although RVS infiltration is anatomically plausible via the peritoneal reflection and has been historically described, including through possible retrograde menstruation or Müllerian metaplasia, its true prevalence remains uncertain and likely overestimated. Additional confusion arises from the broad term ‘rectovaginal endometriosis’ (RVE), often applied to any lesion between the rectum and vagina, even when neither structure is infiltrated. This semantic overlap contributes to the misclassification of lesions in the posterior compartment as ‘RVS’ endometriosis.

## Methods

We conducted a prospective observational study at the McMaster University Endometriosis Clinic from November 2021 to January 2023 to determine endometriosis distribution with a modern approach, combining ultrasound, surgery, and histopathology, and conforming to the latest International Terminology definitions of DE as any lesion with infiltration depth below the peritoneal surface (replacing the former >5 mm threshold). Patients undergoing surgery, and respective histopathological confirmation, for pelvic pain and/or infertility were included and assessed for all forms of endometriosis, with a focus on ovarian endometriosis (OE) and DE, as these subtypes are felt to be reliably diagnosed/ruled out with a combination of TVS and surgery. Endometriosis was described as present or absent in the following locations: ovarian, uterosacral ligaments (USL), torus uterinus (TU)/RUP peritoneum, vaginal, parametrium, rectum/rectosigmoid, pararectal space, and RVS.

## Results

None of the 161 patients in our study exhibited RVS endometriosis, neither during TVS nor surgery. The prevalence rates were OE (46.3%) and DE of USL/TU/RUP peritoneum (52.8%), rectum/rectosigmoid (19.9%), pararectal space (18.0%), vaginal (13.0%), and parametrium (5.6%). RUP obliteration was noted in 27.9% (45/161) of participants. DE was often identified upon dissection of adhesions, giving the impression of being in a ‘retroperitoneal location’. In all cases, the RVS was assessed as preserved, with no evidence of DE.

## Conclusion

We observed no cases of RVS DE despite systematic sonographic and surgical assessment, contrasting with historically reported rates (∼40–50%). Our findings suggest that RVS DE is uncommon and likely overdiagnosed, based on misconceptions of anatomy and/or terminology. It is most likely that lesions at the peritoneal reflection (the most cephalad aspect and ‘surgical’ entry to the RVS), with or without significant inferior infiltration, have been described as RVS lesions, with lower rectal DE (<7 cm) extending into the retroperitoneum, close to the RVS (Szabó *et al.* 2024). We do not doubt that RVS DE exists, as endometriosis does not respect anatomical borders (Szabó *et al.* 2022); instead, we believe modern TVS approaches, adapted from the IDEA consensus, and detailed minimally invasive surgical mapping can better differentiate intraperitoneal lesions, often at the USL, TU, or RUP peritoneum, or at the anterior rectal wall, from true retroperitoneal RVS involvement. Precise terminology and accurate localisation remain critical for improving diagnostic consistency in endometriosis. The IDEA consensus recommends using ‘posterior compartment’ to describe the area behind the uterus, including the rectum, vagina, USLs, and RUP. The term RVE should be retired. We must avoid falsely labelling USL, TU, or RUP peritoneal lesions as RVS, using cues such as pelvic fluid, cervical positioning, and the understanding that endometriosis originates as an intraperitoneal disease to distinguish what is above the peritoneal reflection from what is within the RVS.

## Declaration of interest

M Leonardi is an Associate Editor of *Reproduction & Fertility* and was not involved in the review or editorial process for this paper, on which he is listed as an author. M Leonardi reports grants from Australian MRFF, AbbVie, CanSAGE, Hamilton Health Sciences, Hyivy, and Pfizer; honoraria for lectures/writing from AIUM, GE Healthcare, Bayer, AbbVie, TerSera, Samsung, and Canon; consultancy work with Hologic, Chugai, and Roche Diagnostics; affiliations with IMAGENDO, outside the submitted work. G Condous reports grants from Australian MRFF, ASUM, and Endometriosis Australia; honorarium from GE Healthcare and Samsung; and affiliation with IMAGENDO, outside the submitted work. S M Freger reports advocacy work with TENC and EndoACT. The remaining authors have no conflicts of interest to report.

## Funding

This research did not receive funding from public, commercial, or not-for-profit sector agencies.

## Author contribution statement

All authors contributed equally throughout the study and the development of this manuscript.

## Ethics approval

The Hamilton Integrated Research Ethics Board reviewed and approved this study on September 6th, 2020, as part of HiREB: 12617.

## References

[bib1] Guerriero S, Ajossa S, Gerada M, et al. 2008 Diagnostic value of transvaginal “tenderness-guided” ultrasonography for the prediction of location of deep endometriosis. Hum Reprod 23 2452–2457. (10.1093/humrep/den293) 18664469

[bib2] Guerriero S, Condous G, van den Bosch T, et al. 2016 Systematic approach to sonographic evaluation of the pelvis in women with suspected endometriosis, including terms, definitions and measurements: a consensus opinion from the International Deep Endometriosis Analysis (IDEA) group. Ultrasound Obstet Gynecol 48 318–332. (10.1002/uog.15955) 27349699

[bib3] International Working Group of AAGL, ESGE, ESHRE and WES, Tomassetti C, Johnson NP, Petrozza J, et al. 2021 An international terminology for endometriosis, 2021. Hum Reprod Open 2021 16. (10.1093/hropen/hoab029) PMC8530702 34693033

[bib4] Nisenblat V, Bossuyt PMM, Farquhar C, et al. 2016 Imaging modalities for the non-invasive diagnosis of endometriosis. Cochrane Database Syst Rev 2016 CD009591. (10.1002/14651858.cd009591.pub2) PMC7100540 26919512

[bib5] Szabó G, Madár I, János R, et al. 2022 A novel complementary method for ultrasonographic screening of deep endometriosis: a case series of 5 patients diagnosed with transvaginal strain elastography. Clin Exp Obstet Gynecol 49 1. (10.31083/j.ceog4901002)

[bib6] Szabó G, Hudelist G, Madár I, et al. 2024 Diagnostic accuracy of the IDEA protocol for non invasive diagnosis of rectosigmoid DE – a prospective cohort study. Ultraschall Med 45 61–68. (10.1055/a-2034-2022) 36781162

